# Drought Tolerance in Wheat

**DOI:** 10.1155/2013/610721

**Published:** 2013-11-11

**Authors:** Arash Nezhadahmadi, Zakaria Hossain Prodhan, Golam Faruq

**Affiliations:** Institute of Biological Sciences, Faculty of Science, Universiti Malaya, 50603 Kuala Lumpur, Malaysia

## Abstract

Drought is one of the most important phenomena which limit crops' production and yield. Crops demonstrate various morphological, physiological, biochemical, and molecular responses to tackle drought stress. Plants' vegetative and reproductive stages are intensively influenced by drought stress. Drought tolerance is a complicated trait which is controlled by polygenes and their expressions are influenced by various environmental elements. This means that breeding for this trait is so difficult and new molecular methods such as molecular markers, quantitative trait loci (QTL) mapping strategies, and expression patterns of genes should be applied to produce drought tolerant genotypes. In wheat, there are several genes which are responsible for drought stress tolerance and produce different types of enzymes and proteins for instance, late embryogenesis abundant (lea), responsive to abscisic acid (Rab), rubisco, helicase, proline, glutathione-S-transferase (GST), and carbohydrates during drought stress. This review paper has concentrated on the study of water limitation and its effects on morphological, physiological, biochemical, and molecular responses of wheat with the possible losses caused by drought stress.

## 1. Introduction

Drought is one of the most common environmental stresses that affect growth and development of plants. Drought continues to be an important challenge to agricultural researchers and plant breeders. It is assumed that by the year 2025, around 1.8 billion people will face absolute water shortage and 65% of the world's population will live under water-stressed environments. Tolerance to water stress is a complicated parameter in which crops' performance can be influenced by several characteristics [1]. Tolerance can be divided into two parts including drought avoidance and dehydration tolerance [2]. Drought avoidance includes root depth, reasonable use of available water by plants, and changes in plants' lifestyle to use rainfall. Dehydration tolerance consists of plants' capability to partially dehydrate and grow again when rainfall continues [3]. Adaption of plants to drought stress is a vital issue to develop new improve methods for increasing stress tolerant plants [4]. Many factors can affect plants' responses to drought stress such as plant genotype, growth stage, severity and duration of stress, physiological process of growth [5], different patterns of genes expression [6], different patterns of the activity of respiration [7], activity of photosynthesis machinery [8], and environmental factors [4, 9]. Drought stress can have effects on genes expression, and detection of genes during water stress is crucial to observe their responses. In this regard, various drought responsive genes were distinguished [1]. Ouvrard et al. [10] believed that the role of genes can be distinguished by expression of a gene to high resistance levels among varieties. Drought stress can also influence plants in terms of protein changes, antioxidant production, osmotic adjustment, hormone composition, root depth and extension, opening and closing of stomata, cuticle thickness, inhibition of photosynthesis, decrease in chlorophyll content, reduction in transpiration, and growth inhibition [11–14] to stand with some osmotic changes in their organs. Drought can also cause pollen sterility, grain loss, accumulation of abscisic acid in spikes of drought-susceptible wheat genotypes, and abscisic acid synthesis genes in the anthers [15]. In many biochemical studies, the role of reactive oxygen species (ROS) has been identified. Dat et al. [16] claimed that increase in ROS can be caused by drought stress in which oxidative balance of the cell is changed. A rise in the generation of ROS prompts to the generation of ABA (abscisic acid) which is a general signal under drought [17–20] and can consequently regulate the antioxidant genes expressions by producing superoxide dismutase (SOD) and catalase (CAT) [21]. Several physiological studies have been completed on the impact of drought stress on wheat. Rosenberg et al. [22] observed that transpiration decreased significantly under drought stress; then heat can slowly be lost from the leaves and leaf temperature can be increased. As a result, CO_2_ concentrations and photosynthesis are increased which affect plant's growth and finally, water use efficiency can be improved. The same studies demonstrated that plants' development can be promoted more with CO_2_ [23–26]. A respiratory terminal oxidase, alternative oxidase (AOX), plays important roles in optimizing photosynthesis and protecting chloroplast under drought stress [27]. Ribas-Carbo et al. [7] suggested that the increase in AOX pathway under water stress could be prompted by the inhibition of cytochrome pathway. In this review paper, an attempt is made to explore different research information on wheat drought tolerance in various aspects, namely, morphological, physiological, biochemical, and molecular responses.

## 2. Physiological Derivations of Drought Tolerance in Wheat

Physiological responses include closure of stomata, decrease in the activity of photosynthesis, development of oxidative stress, alteration in the integrity of cell wall, production of metabolites which are toxic and cause plants' death [28], signal recognition of roots, turgor loss and adjustment of osmosis, reduction in water potential of leaf, decrease in stomata conductance to CO_2_, reduction of internal CO_2_ concentration, and reduction of growth rates. According to researchers, there is a relationship between different physiological responses of crops and their resistance functions under drought such as high amount of relative water and potential water [29, 30] and integrity of membrane [31, 32]. For measuring drought tolerance, various scientists considered maintenance of membrane integrity and its role under water stress [33, 34]. Sink strength can be reduced in drought stress during early grain filling which results in reducing endosperm cell number and metabolic activity [35]. Grudkowska and Zagdańska [36] indicated that cysteine proteinase plays an imperative function in plant signalling pathways, growth and development, and in the response to various kinds of stress. Cysteine is expressed in wheat leaf organs and its contribution in proteolysis activity rises under drought [37]. Wiśniewski and Zagdańska [38] also observed that the role of cysteine was improved, but its role was negatively related to the degree of drought tolerance of ten lines of spring wheat. Transpiration efficiency (TE) is indispensable phenomenon in plants. Various researchers proposed that TE can be influenced by cultivar and drought [39, 40]. So, the selection of high TE crops is the most important action to produce drought tolerant plants. Growth is one of the physiological processes which is sensitive to drought and can be affected by reduction in turgor pressure. Because of low turgor pressure, water stress quenches cell expansion and growth. However, when turgor pressure is bigger than the cell wall yield, cell expansion can occur [41, 42]. Osmotic adjustment is a remarkable part of plants' physiology by which they respond to water deficits [5, 43–47]. Yield losses at vegetative growth and reproductive stages under drought in wheat are provided in Tables 1 and 2.

## 3. Biochemical Derivations of Drought Tolerance in Wheat

A reduction in efficiency of photochemical, reduced Rubisco efficiency, gathering of stress metabolites (glutathione, MDHA, glybet, and polyamines), antioxidative enzymes (superoxide dismutase (SOD), peroxidase (POD), catalase (CAT), ascorbate peroxidase (APX), glutathione reductase (GR), glutathione-S-transferase (GST), glutathione peroxidase (GP)), monodehydroascorbate reductase (MDHAR), and reduced ROS accumulation are biochemical responses of plants to water stress. Tolerance to drought correlates with a positive response of plants' antioxidant system. According to the study of Li and Staden [55], in drought condition, some reactive oxygen species (ROS) such as hydroxyls (OH), superoxide (O_2_^−^), peroxide hydrogen (H_2_O_2_), and oxygen which is singlet (^1^O_2_) are created. These ingredients may initiate disturbing lipid peroxidation, chlorophyll, protein oxidation, and nucleic acids [56]. Changes in activity of these enzymes are crucial for the resistance of various plants to drought stress [57]. Evidences suggest that drought causes oxidation damage from increased production of ROS with deficit defense system of antioxidant in plants [54, 58–61]. Osmotic regulators include small molecules (Pro), ions (K+), and soluble sugar, which help crops to absorb water in drought environments. In wheat, various studies exhibited that wheat genotypes with higher osmotic regulators and lower malondialdehyde (MDA) content have better tolerance to drought [5, 43, 47, 60, 62–66]. Polyamines (PAs) have a role in the completeness of membranes and nucleic acid under water stress environments [11]. Malabika and Wu [67] mentioned that higher levels of polyamines can make crops have higher growth under water stress conditions [18, 68, 69]. CAT is one of the most rapidly reversible proteins in leaf cells especially in stress conditions and its activity is reduced in drought condition [70]. 

## 4. Morphological Derivations of Drought Tolerance in Wheat

According to the study of Deňcić et al. [71], wheat is paid special attention due to its morphological traits during drought stress including leaf (shape, expansion, area, size, senescence, pubescence, waxiness, and cuticle tolerance) and root (dry weight, density, and length). Shi et al. [72] expressed that drought can affect vegetative and reproductive stages. Therefore, understanding plants' responses to drought at every life stage is crucial to progress in genetic engineering and breeding. Rizza et al. [50] observed that early maturity, small plant size, and reduced leaf area can be related to drought tolerance. Lonbani and Arzani [73] claimed that the length and area of flag leaf in wheat increased while the width of the flag leaf did not significantly change under drought stress. Leaf extension can also be limited under water stress in order to get a balance between the water absorbed by roots and the water status of plant tissues [74]. According to the study of Rucker et al. [75], drought can reduce leaf area which can consequently lessen photosynthesis. Moreover, the number of leaves per plant, leaf size, and leaf longevity can be shrunk by water stress [76]. Singh et al. [53] observed that leaf development was more susceptible to water stress in wheat. Root is an important organ as it has the capability to move in order to find water [77]. It is the first organ to be induced by drought stress [78]. In drought stress condition, roots continue to grow to find water, but the airy organs are limited to develop. This different growth response of shoots and roots to drought is an adaptation to arid conditions [79, 80]. To facilitate water absorption, root-to-shoot ratio rises under drought conditions [81, 82] which are linked to the ABA content of roots and shoots [83]. The growth rate of wheat roots was diminished under moderate and high drought conditions [84]. In wheat, the root growth was not markedly decreased under drought [85]. Plant biomass is a crucial parameter which was decreased under drought stress in spring wheat [86]. The same outcomes were observed in previous studies in wheat and other crops [86–88]. In winter wheat, the yield was decreased or changed under drought and, in contrast, the water use efficiency was boosted [89, 90].

## 5. Molecular Responses of Drought Tolerance in Wheat

Some genes are known to be drought influenced and produced different types of drought stress related proteins and enzymes including dehydrins [91], vacuolar acid invertase [92], glutathione S-transferase (GST) [93], and late embryo abundant (LEA) protein [94]; expression of *ABA* genes and production of proteins like RAB, rubisco, helicase, proline, and carbohydrates are molecular basis of drought tolerance. Plants respond to stress environments with altering their gene expressions and protein productions. In contrast, available information on drought-responsive genes is still limited as their roles have not been thoroughly determined [28]. In wheat seedling stage, a lot of studies are done in gene expression, but it is the junction stage that is susceptible to drought [72]. This is because junction phase is the linkage point in the vegetative and flowering growth stages and it is important for development and reproduction [72]. Sivamani et al. [52] indicated that *HVA1* gene assists to increase wheat growth under drought stress. *HVA1* gene produce a kind of protein which is in group 3 LEA and has 11 amino acid motifs in nine repeats. Proline is a crucial protein that has a vital function in water stress tolerance. It can be created from pyrroline-5-carboxylate synthetase or P5CR, and the responsible gene for this enzyme has been distinguished in some crops, namely, petunia, soybean, and tobacco [95–97]. Hong-Bo et al. [98] investigated the role of proline as a wheat antidrought defence protein under drought. In photosystem II (PS II) reaction center, psbr has an indispensable task in oxidation of water [99], and in Calvin cycle, rubisco is the key enzyme under drought stress [100]. Some plant proteins can be over-expressed including late embryogenesis abundant (LEA) that are saved in vegetative tissues during desiccation of seeds under drought stress. LEA proteins are influenced by drought stress and their size in wheat reaches 200 kDa (Wcs200) [101]. These proteins have been detected through their sequence of amino acid [102] and they help other proteins retrieve after denaturation during water stress [103]. There have been a lot of works during the last two decades to engineer LEA producing genes for promoting crop water stress resistance. For instance, wheat LEA genes, PMA1959 (encoding group one of LEA protein) and PMA80 (encoding LEA protein's second group) improved water deficit resistance in rice [104]. In wheat, protein contents of groups one, two, and three of LEA have been detected. The *Em* gene of wheat which encodes LEA protein first group has been vastly researched [105–107]. Group three of LEA protein has also been distinguished in seedlings of wheat [108, 109]. In durum wheat, protein of groups two (dehydrins) and four of LEA proteins were studied by Ali-Benali et al. [110]. *Td27e*, *Td29b*, and *Td16* gene transcripts were saved late in embryogenesis and throughout seed development. Transcripts of *Td11* gene were presented whereas no transcripts of *Td25a* gene were detected in seeds [110]. Vacuolar H^+^-translocating pyrophosphatase (V-PPase) is an important enzyme linked to plant development as well as resistance to abiotic stress. Wheat V-PPase genes, *TaVP3*, *TaVP2*, and *TaVP1* were investigated by Wang et al. [111]. Kam et al. [112] also detected the responsible genes in wheat for water stress. They observed that *TaRZF70* as a RING-H2 zinc finger gene presented various responses to drought stress which was upregulated in the leaf and downregulated in the root [113]. *TaRZF38* and *TaRZF70* were expressed in the wheat root while *TaRZF74* and *TaRZF59* were expressed in embryo and endosperm at the highest level. TACCGACAT, the 9-bp consensus sequence, was first distinguished in the promoter of Arabidopsis *rd29A/lti78* and presented to be vital for drought induction in abscisic acid absence [114]. Then, this element could be bent by a family of transcription elements and therefore named DRE-binding (DREB) proteins [115]. Lucas et al. [116] used a sequence of putative DREB labelled DREB3A from wheat (TaDREB3A, Gen bank ID: AY781349) to seclude a DREB from wild wheat (*T. turgidum *ssp*. dicoccoides*) and to detect its function in higher drought resistance. They also concluded that DREB proteins are numerous and vastly upregulated in reaction to drought in root tissue rather than leaf [116]. Drought stress influences *RD* gene (responsive to desiccation) [117, 118]. This gene has been divided into two major parts. The first group includes expression of regulatory gene and signal direction during the crops' reaction to stress, and the second group involves proteins which directly protect cells from stresses [119]. In wheat, among 265 genes detected at the junction phase and 146 genes distinguished at the seedling stage in response to drought stress, more than half of them were thought to be involved in abiotic or biotic stress responses [72]. 

## 6. Breeding for Drought Tolerance through Conventional and Biotechnological Breeding Methods

Conventional breeding needs the detection of genetic variability under drought between plant genotypes, or between sexually compatible cultivars, and introduction of tolerance line with proper agronomic traits. Although conventional breeding for water stress resistance has had some prosperity, it is a slow process which is limited by the availability of proper genes for breeding. In traditional breeding, crosses are partially uncontrolled and breeders select parents to cross, but at the genetic approach, the outcomes are unpredictable [120]. Conventional breeding strategies are labour-intensive which requires great efforts to separate undesirable traits from desirable traits, and this is not economically suitable. For instance, crops must be back-crossed again over lots of growing seasons to breed undesirable traits generated by random mixing of genomes [120]. On the other hand, the improvement of resistant plants through genetic engineering needs detection of important genetic dominants to respond as stress resistance crops by transferring novel genes into plants. Drought affects the activity of a vast number of genes, and gene expression experiments have detected various genes that are induced and repressed under drought stress [121]. The nature of drought tolerance makes the management difficult in traditional breeding techniques. Novel biotechnological strategies have increased information on crop responses to drought at whole crop and molecular levels [122]. A lot of drought stress-induced genes were detected and cloned. Crop genetic engineering and molecular-marker methods make the improvement of drought-resistant germplasm possible [122]. Transgenic crops are also being improved to manage water stress. Structural and regulatory genes including dehydration-responsive, element-binding (DREB) factors, zinc finger proteins, and NAC transcription factor genes are already being applied [122]. Agrobacterium and particle gun techniques for transgenes related to drought resistance were applied in different crops such as rice, wheat, maize, sugarcane, tobacco, Arabidopsis, groundnut, tomato, and potato. Drought-tolerant genetically modified (GM) plants are being produced and molecular markers are used to detect drought-related quantitative trait loci (QTL) which were successfully transferred into rice, wheat, maize, pearl millet, and barley [122].

## 7. Breeding for Drought Tolerance through Molecular Markers in Wheat

Nowadays, molecular markers are widely used to detect the location of drought-induced genes. Different molecular marker are currently available for genome mapping and tagging of different traits which is useful for Marker-assisted breeding (MAB) technique in wheat in stress conditions [123]. It is intensively used to create stress-tolerant lines in different crops. Marker-assisted selection (MAS) refers to selection by DNA markers linked to QTLs that are very powerful. Thus, DNA markers can track presence of QTLs for drought tolerance [124, 125]. For development of drought tolerance in plants through molecular linkage maps, marker-assisted selection (MAS) is the best procedure. In winter wheat, with the use of amplified fragment length polymorphism (AFLP) and simple sequence repeat (SSR) markers, QTL mappings for senescence of flag leaf (FLS) in normal and water-stressed environments have been studied. The responsible gene for this characteristic is revealed and the QTL is also detected on chromosome 2D associated with better performance under drought [126]. In another study by Quarrie et al. [127], DNA markers like restriction fragment length polymorphism (RFLP), AFLP, and SSR have been used to tag QTLs for drought stress in wheat. During the last few decades, molecular markers such as SDS-protein, isozymes, and DNA sequences have assisted to select quantitative traits especially drought tolerance. These molecular markers are used in wheat to evaluate diversity of genes and identify genotype and genetic mapping [128–130]. Some markers in durum wheat are linked to grain yield and morphophysiological characteristics for drought tolerance [131]. Leaf water potential, canopy temperature, chlorophyll inhibition, and proline content showed strong relationships with molecular markers [131]. Ashraf et al. [132] prepared various DNA markers to estimate inheritance of stress tolerance such as PCR indels, RAPDs, RFLPs, CAPS, AFLPs, microsatellites (SSRs), SNPs and sequences of DNA. In cereals, RAPDs with the use of DNA primer were vastly used [133, 134]. ISSRs were used in mapping of genome in wheat and other crops [135, 136]. Milad et al. [48] identified RAPD and ISSR markers related to flag leaf senescence gene in wheat under drought stress. RAPDs were found to be helpful in hexaploid wheat as genetic markers [134, 137]. When the correlation between a molecular marker and a trait is greater than the heritability of the trait, marker assisted selection may be advantageous. These results suggest the usefulness of molecular markers to enhance drought tolerance in durum wheat in drought condition [131]. 

## 8. Mapping of QTL for Drought Tolerance in Wheat

Quantitative trait loci (QTL) is a location from where some genes influence a phenotype of quantitatively inherited trait. Genetic variations of a crop can be explored through QTL mapping (polygenes) [132]. Mapping of QTL allows the estimation of the places, quantity, size of effects for the phenotype, and gene activity pattern [138]. In 2005, the first activity was conducted for cloning QTL [139] to know and operate the characteristics which are responsible for drought resistance [51, 140, 141]. QTL mapping for water stress resistance traits has been done in wheat and other crops [142–147]. In wheat, due to drought stress, the place of genes which had influence on ABA concentration was detected [142]. It is detected that 5A chromosome transports gene(s) for ABA concentration. Quarrie et al. [127] conducted mapping of QTLs for drought resistance in hexaploid wheat placed on chromosomes 1A, 1B, 2A, 2B, 2D, 3D, 5A, 5B, 7A, and 7B. Double haploid populations serve as a permanent source of QTL mappings. Recombinant inbred lines from crossing of drought-resistant and drought-susceptible cultivars were used to create mapping populations for QTL analysis regulating yield under drought [148]. QTL analysis is so important to target genes and for doing this some steps are required. Firstly, phenotypic evaluation of relatively large population for markers which are polymorphic is needed. Secondly, genotyping of the population is important. Thirdly, there is a need for statistical analysis to detect the loci that are influencing the target trait. On the other hand, QTL for drought tolerance has some drawbacks like genetic and environmental interactions, numerous numbers of genes, and using of mapping populations which are wrong. These have limited plans for mapping of QTL for high yield under drought condition [149]. 

## 9. Drought Management

### 9.1. Drought-Tolerant Varieties

In the past decade, there have been several efforts to generate drought-tolerant wheat through breeding methods. Cross-breeding among wild wheat species at the International Centre for Agricultural Research in the Dry Areas (ICARDA) created germplasm that creates higher yields under drought. In wheat breeding programs, seeking for increased yield has been a priority to improve drought tolerance of plants. However, before successful genetic manipulation can be made, it is important to characterize the physiological parameters of drought-tolerant or -sensitive cultivars [150]. Analysing physiological determinants for yield which responds to water stress may also be helpful in breeding for higher yields and stability of genotypes under drought conditions. Traits to select either for stress escape, avoidance or tolerance, and the framework where breeding for drought stress is addressed will depend on the level and timing of stress in the targeted areas. However, selecting for yield itself under stress-alleviated conditions appears to produce superior cultivars, not only for optimum environments, but also for those characterized by frequent mild and moderate stress conditions [150]. This implies that broad avoidance/tolerance to mild/moderate stresses is given by constitutive traits also expressed under stress-free conditions [151]. Keeping in view the importance of identifying water-stress tolerant wheat genotypes, water stress conditions can be imposed to wheat at various stages of crop growth and development. The stresses can be given at tillering, booting, and grain forming stages. Root system size (RSS) of wheat can be a selection target for drought tolerance. During dry periods, crops expand their roots to deeper soil regions and they are able to alter their morphology. For instance, the airy organ mass is decreased but the mass of roots is increased. Wheat genotypes with good water management are able to bear high yields in drought conditions [152]. Genotypes with proper water management could be used to create new breeding lines and cultivars with developed drought resistance.

### 9.2. Agronomic Practices

**Figure 1:**
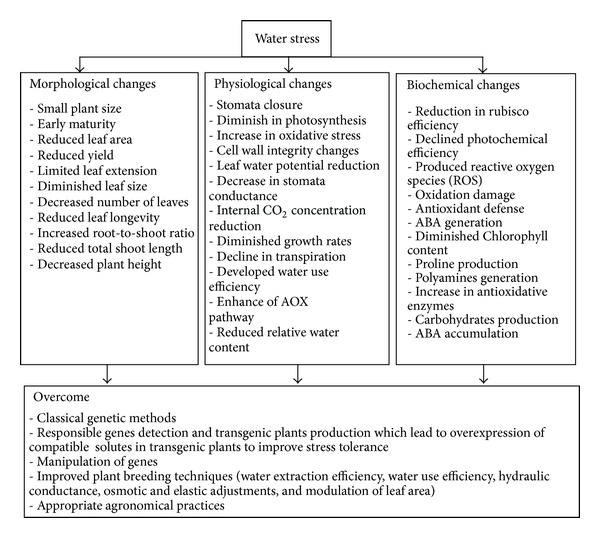
The effect of drought stress on wheat. The information are provided from the observations of Powel et al. [128], Lawlor and Cornic [13], Shiran and Wan [15], Karthikeyan et al. [41], and Russell et al. [129].

Drought stress includes different agronomic, soil, and climatic factors which vary in the time of occurrence, duration, and intensity. It has effect on yield and can also diminish benefits of crop handling performances including management of fertilizer or pest and disease [49]. Drought management strategies are very important and have to concentrate on extraction of available soil moisture, crop establishment, growth, biomass, and grain yield. There are many agronomical ways to manage drought stress such as control of field irrigation methods (surface or furrow, sprinkled, and drip) and identification of drought resistance sources through developing screening methods under environmental conditions. So, for drought screening, not only analysing sources of replications, variation among plots, and repeated experiments are needed, but also sprinkler irrigation, rainout shelters, and evaluation of drought susceptibility index (DSI) are important [49]. In drought management strategies, increasing biomass and seed yield, crop establishment, and maximum crop growth have to be considered. For example, to improve yield in drought-prone area, these steps are essential: frequency of drought stress occurrence in the target environment, matching phenology of crop (sowing, growth period, flowering, and seed filling) with period of soil moisture and climatic regimes, developing a way for the better use of irrigation, and increasing soil water to crop through agronomic management practices. Furthermore, good knowledge of what type of stress is more frequent in target environment is essential in drought breeding. Yield stability under water shortage condition and crop water productivity should be the goal. In drought stress condition, the aim is to preserve the source of water. These sources include snow, rain, and irrigation water. Water conservation can be achieved by surface residue during the growing season. Todd et al. [163] claimed that wheat residue diminished the evaporation rate during the season. Residue also slows movement of water and allows much time for the water to penetrate into the soil. Rotation of crop can preserve the total water needs by irrigation. In winter wheat, it can be decline requirements for irrigation. Schneekloth et al. [159] claimed that with irrigation for 6 inches, corn following wheat produced 8 percent more than corn following corn. Rotation of crops also makes the irrigation season to have much time frame in comparison with a single crop. In breeding for drought resistance, productions of biomass and water use efficiency (WUE) are imperative elements of agronomy [155]. There is a risen interest in improving WUE of plant genotypes so that plants can develop and bear better under drought condition [154, 164].  Figure 1 shows the effects of drought stress on different wheat traits. Detailed information on physiological, molecular, biochemical, and morphological traits under drought stress in wheat is demonstrated in Tables 3, 4, 5, and 6. 

## 10. Conclusion

Detection of genomic responses of plants to water stress is so important. Firstly, it prepares intensive information about transcriptional reactions of plants to drought stress. Secondly, it makes possible to know functions of genes in stress environments. Thirdly, it assists to distinguish promoters which react to stress and related cis-elements, which are both crucial for primitive studies and crop engineering [165]. Rapid improvements can be performed in drought resistance by manipulating the genes which are responsible for the plant growth regulators, antioxidants, proteins, and transcriptional factors [149]. QTL analysis and molecular mapping are also proper methods which have been done for qualitative and quantitative characteristics including resistance for stress. But, there are some limitations in this issue. For example, there is a challenge for QTL detection, for instance, interaction between genotype and environment, inconsistent repeatability, numerous genes that regulate yield, and use of wrong populations for mapping. Furthermore, other elements also limit the efficiency of QTL for genetic development of a parameter because of improper interaction epistasis, it is difficult to carry the influences of an allele to extract substance [156, 166]. Moreover, in several circumstances, QTL does not present marked impacts and stop thoroughly in various groundwork, even in similar growth conditions [153, 156]. This high variability in the nature of water stress and inadequate information about its complicatedness have caused it to be hard to identify specific physiological traits needed for improved crop performance.

## Figures and Tables

**Table 1:** Yield losses at vegetative growth stages under drought in wheat.

Vegetative stage	Yield loss (%)	Reference
Early season stress	22	[48]
Midseason stress	58	[48]
Booting stage	20.74	[49]
Tillering stage	46.85	[49]
1000-grain weight (vegetative stage)	38.67	[50]
Earlier stages	79.7	[51, 52]
Spike length (vegetative stage)	16.90	[50]
Number of spikelets per spike (vegetative stage)	28.63	[50]
Grains number (vegetative stage)	72.51	[50]
Grain yield (vegetative stage)	61.38	[50]

**Table 2:** Yield losses at reproductive growth stages under drought in wheat.

Reproductive stage	Yield loss (%)	Reference
Higher grain protein content, fewer days to physiological maturity, smaller kernel weight and diameter, less grain yield	Not applicable	[53]
Less grain yield (drought-tolerant variety)	43	[54]
Less grain yield (drought-sensitive variety)	26	[54]
1000-grain weight	18.29	[1]
	5	[3]
1000-grain weight (anthesis stage)	38.67	[50]
Biological yield	10	[1]
Maximum grain yield	22	[1]
Decreased seed number	64	[3]
Grain formation stage	101.23	[49]
Grain formation stage	65.5	[51, 52]
Number of spikes	19.85	[50]
Number of spikes (anthesis stage)	15.79	[50]
Spike length (anthesis stage)	16.90	[50]
Number of spikelets per spike (anthesis stage)	26.20	[50]
Grains number (anthesis stage)	72.51	[50]
Grain yield (anthesis stage)	64.46	[50]

**Table 3:** Research scenario of physiological traits under drought stress in wheat.

Traits	Reference
Physiological	
Stomata closure	[16]
Cell wall integrity	[16]
Synthesis of metabolites	[16]
Oxidative stress	[16]
Photosynthesis	[16, 45, 62, 115]
Turgor pressure	[64, 71]
CO_2_ concentration	[30, 46, 73, 84]
Growth rate	[92]
Osmotic adjustment	[21, 42, 62, 81, 88, 89, 97]
Stomata conductance	[62]
Relative water content	[26, 110]
Membrane integrity	[36, 72, 102, 119]
Transpiration	[115]
Water use efficiency	[13, 69, 153]
Transpiration efficiency	[121, 124]
Total biomass	[13, 154, 155]
Alternative oxidase (AOX)	[108, 156]

**Table 4:** Research scenario of molecular traits under drought stress in wheat.

Traits	Reference
Molecular	
CAT gene expression	[66]
SOD gene expression	[66]
Proline	[57, 114, 157]
Dehydrins	[2, 27]
Vacuolar acid invertase	[150]
Glutathione S-transferase (GST)	[5]
Late embryo abundant (LEA)	[17, 40, 98]
DRE-binding proteins	[78]
Rd29A/Lti78	[158]
Psbr	[142]
Rubisco	[43]
QTL mapping	[8, 9, 12, 19, 44, 103, 122, 123, 125, 126, 145, 146, 149, 151, 152, 159]
Molecular markers	[9, 44, 146]

**Table 5:** Research scenario of morphological traits under drought stress in wheat.

Traits	Reference
Morphological	
Small plant size	[112]
Leaf area	[112, 116]
Root extension	[75, 135, 143, 160, 161]
Roots dry weight, density, and length	[35]
Early maturity	[112]
Yield	[69, 90, 128, 153]
Leaf extension	[95]
Leaf size	[133]
Leaf number	[133]
Leaf longevity	[133]
Root-to-shoot ratio	[88, 91]

**Table 6:** Research scenario of biochemical traits under drought stress in wheat.

Traits	Reference
Biochemical	
Chlorophyll content	[75, 100, 127, 143, 160, 161]
Superoxide Dismutase (SOD)	[66]
Catalase (CAT)	[59, 66]
Polyamines (PAs)	[4, 14, 82, 136, 143]
Reactive oxygen species (ROS)	[22, 24, 31, 32, 56, 76, 96, 107, 127, 132, 136, 139, 162]
Abscisic acid (ABA)	[32, 56, 96, 136]
